# Comparison of respiratory pathogens in children with community-acquired pneumonia before and during the COVID-19 pandemic

**DOI:** 10.1186/s12887-023-04246-0

**Published:** 2023-10-27

**Authors:** Mingyu Tang, Wenfang Dong, Shuhua Yuan, Jiande Chen, Jie Lin, Jinhong Wu, Jing Zhang, Yong Yin, Lei Zhang

**Affiliations:** Department of Respiratory Medicine, Shanghai Children’s Medical Center, Shanghai Jiao Tong University School of Medicine, 1678 Dongfang Rd, Shanghai, 200127 China

**Keywords:** COVID-19, Respiratory pathogens, Prevalence, Children, Pneumonia

## Abstract

**Background:**

Multifaceted non-pharmaceutical interventions during the COVID-19 pandemic have not only reduced the transmission of SARS-CoV2, but have had an effect on the prevalence of other pathogens. This retrospective study aimed to compare and analyze the changes of respiratory pathogens in hospitalized children with community-acquired pneumonia.

**Methods:**

From January 2019 to December 2020, children with community-acquired pneumonia were selected from the Department of Respiratory Medicine, Shanghai Children’s Medical Center. On the first day of hospitalization, sputum, throat swabs, venous blood samples from them were collected for detection of pathogens.

**Results:**

A total of 2596 children with community-acquired pneumonia were enrolled, including 1871 patients in 2019 and 725 in 2020. The detection rate in 2020 was lower than in 2019, whether single or multiple pathogens. Compared with 2019, the detection rate of virus, especially parainfluenza virus, influenza virus and respiratory syncytial virus, all decreased in 2020. On the contrary, the prevalence of human rhinovirus was much higher than that in 2019. In addition, the positivity rate for bacteria did not change much over the two years, which seemed to be less affected by COVID-19. And *Mycoplasma pneumoniae* which broke out in 2019 has been in low prevalence since March 2020 even following the reopening of school.

**Conclusions:**

Strict public health interventions for COVID-19 in China have effectively suppressed the spread of not only SARS-CoV2 but parainfluenza virus, influenza virus and *Mycoplasma pneumonia* as well. However, it had a much more limited effect on bacteria and rhinovirus. Therefore, more epidemiological surveillance of respiratory pathogens will help improve early preventive measures.

**Supplementary Information:**

The online version contains supplementary material available at 10.1186/s12887-023-04246-0.

## Background

In December 2019, the coronavirus disease 2019 (COVID-19) caused by severe acute respiratory syndrome coronavirus 2 (SARS-CoV-2) broke out in Wuhan, Hubei, China [1]. On 31 January 2020, COVID-19 was declared a Public Health Emergency of International Concern (PHEIC) by the World Health Organization (WHO). According to Johns Hopkins Coronavirus Resource Center, by the end of October 2021, more than 230 million people were infected with this virus, of which 4.8 million had died [2]. COVID-19 is mainly transmitted through contact and droplets, and the population is susceptible in general [3–5]. Since February 2020 [6], China has taken a various of non-pharmaceutical interventions (NPIs) measures to curb the spread of this virus, such as wearing masks, washing hands frequently, paying attention to indoor ventilation, maintaining social distance and supporting employees to work and study at home. Since the outbreak of the epidemic, Shanghai has entered a stage of normalization of epidemic prevention and control. The government postponed the start of the spring semester of 2020 in primary and secondary schools and cancelled all offline training courses. Consequently, during the COVID-19 pandemic, students had to obtain online courses at home until schools reopened in early June 2020. During this time, Shanghai had strict border controls. Those entering through the Shanghai port were transferred shortly to appointed hotels for at least 14 days of quarantine, and if they tested positive for SARS-CoV-19 PCR during this period, they had to be removed to designated hospitals for further treatment. As of December 31, 2020, confirmed cases of new coronary pneumonia in Shanghai amounted to 349 which were indigenous cases and 1167 which were imported cases.

Lower respiratory tract infections (LRTIs), for instance, bronchiolitis and pneumonia, remain a dominant public health problem and a major cause of morbidity and mortality in children under 5 years old [7]. Since common childhood respiratory pathogens such as respiratory syncytial virus (RSV) and *mycoplasma pneumoniae* (*M. pneumoniae*, MP), share similar routes of transmission with SARS-CoV2, these multifaceted NPIs not only diminish the spreading of the COVID-19, but influence the epidemiology of common childhood respiratory pathogens to a certain extent [8]. In this paper, we aimed to observe the epidemiological characteristics of ordinary respiratory pathogens in children with community-acquired pneumonia (CAP) in 2020 (post-pandemic) and 2019 (pre-pandemic) in Shanghai, China.

## Methods

### Study population

We conducted a retrospective study of children aged 1 month to 16 years with radiologically confirmed community-acquired pneumonia. Venous blood, throat swab and sputum specimens were obtained from these patients on the day of hospitalization at the Department of Respiratory Medicine, Shanghai Children’s Medical Center (SCMC) from January 1, 2019, to December 31, 2020. These specimens were tested for particle agglutination (PA), real-time polymerase chain reaction (RT-PCR), simultaneous amplification and testing (SAT), and bacterial culture of sputum. The study was approved by the Institutional Review Board and the Ethics Committee of Shanghai Children’s Medical Center (SCMCIRB-K2019060-1), and written informed consent was obtained from the parents of each patient.

### Particle agglutination (PA)

Particle agglutination antibody titres for *mycoplasma pneumoniae* were assayed using SERODIA MYCO-II (Fuji Rebio Ltd., Tokyo, Japanese), which was performed using artificial gelatine particles sensitized with cell membrane components of *M. pneumoniae*. The result was considered positive if the titre was 1:160 or more (≥ 1:160).

### Real-time polymerase chain reaction (RT-PCR)

The respiratory secretions of the patient’s throat were collected, sealed and sent for testing by using RT-PCR. The detection reagents for *Mycoplasma pneumoniae* and Legionella pneumophila were provided by Shanghai Zhijiang Biotechnology Co., Ltd. And human rhinovirus (HRV) detection reagents were provided by Hubei Langde Medical Technology Co., Ltd.

### Simultaneous amplification and testing (SAT)

In a short period of time, throat swab samples were collected to identify seven common respiratory pathogen RNAs, including influenza A, influenza B, respiratory syncytial virus (RSV), human parainfluenza virus (HPIV), adenovirus (ADV), *Mycoplasma pneumoniae* (MP) and *Chlamydia pneumoniae* (CP), based on the double-amplification method of RNA isothermal amplification and multiple biotin signals (Zhongzhi Biotechnologies, Wuhan, China).

### Bacterial culture of sputum

On the day of hospitalization, samples were collected using a sterile suction tube attached to a special suction device at one end and the other end inserted into the child’s nasal cavity, from the nasopharynx into the airway, using negative pressure suction to draw sputum out of the respiratory tract. Sputum would be sent to the examination room for screening and pre-treatment before inoculation for culture. The bacterial species mainly linked to community-acquired pneumonia in clinical practice were selected as the target bacterial species, which included *Streptococcus pneumoniae*, *Staphylococcus aureus*, *Escherichia coli*, *Haemophilus infuenzae*, *Pseudomonas aeruginosa*, *Klebsiella sp*, *Acinetobacter sp*, etc.

### Statistical analysis

SPSS software package v25.0 was used for all statistical analyses. Categorical variables were expressed as frequencies and percentages. Proportions of categorical variables were compared using the chi-square test or Fisher’s exact test. P < 0.05 was considered statistically significant.

## Results

### General description

A total of 2596 patients diagnosed with community acquired pneumonia, aged 1 month to 16 years, were registered in the present study, 1871 in 2019 and 725 in 2020. We divided the children into three groups on the basis of age, as follows: infants (age: < 3 years), preschoolers (age: 3–5 years) and school-aged children (age: 6–16 years). Except for the prevalence of infants, which was higher in 2020 than in 2019, the prevalence of preschoolers and school-age children decreased. The proportion of patients with underlying diseases, especially congenital heart disease, and children with severe pneumonia requiring oxygen in 2020 were significantly higher than in 2019. In addition, there was no significant difference in gender, liver function damage, myocardial ischemia and other complications between the year of 2019 and 2020 (Table 1).

**Table 1:** General characteristics of the patients

Characteristic	2019 N = 1871(%)	2020 N = 725(%)	X^2^	P-value
Gender				
Male	966(51.63)	373(51.45)	0.007	0.934
Female	905(48.37)	352(48.55)		
Age of onset				
< 3 years	871(46.55)	417(57.52)	25.129	< 0.001
3–5 years	505(27.00)	167(23.03)	4.263	0.039
6–16 years	495(26.46)	141(19.45)	12.893	< 0.001
Underlying diseases	335(17.90)	253(34.90)	86.109	< 0.001
Congenital heart disease	242(12.93)	174(24.00)	47.547	< 0.001
Brain disease	25(1.34)	17(2.34)	3.340	0.068
Need of oxygen	135(7.22)	87(12.00)	15.296	< 0.001
Complications	151(8.07)	76(10.48)	3.81	0.051
positive pathogens	1451(77.55)	406(56.00)	119.19	< 0.001
single pathogen	1082(57.83)	325(44.83)	35.587	< 0.001
more than two pathogens	369(19.72)	81(11.17)	26.655	< 0.001

In 2019, of 1871 specimens, 1451 (77.55%) tested positive for at least one of the pathogens; among 1082 (57.83%) of these positive specimens, single pathogen was investigated, and in 369 (19.72%) patients, two or more pathogens were detected. In 2020, of 725 specimens, 406 (56.00%) tested positive for at least one of the pathogens; among 325 (44.83%) of these positive patients, single pathogen was investigated, whereas in 81 (11.17%) of patients, there were two or more pathogens. The detection rate in 2020 was substantially lower than that in 2019 (Table 1).

### Comparison of positives rates of pathogens between 2019 and 2020

The top three viruses in 2019 and 2020 were RSV, HRV and HPIV. Except for the significant decrease in the detection rate of HPIV, other viruses showed similar detection rates between the two years. In terms of bacteria, the detection rate of *Haemophilus influenzae* was 7.00% in 2019, but decreased to 1.93% in 2020. In contrast, the detection rates of *Staphylococcus aureus* and *Escherichia coli* in 2020 increased compared with those in 2019. In addition, *Mycoplasma pneumoniae*, one of the most common cause of community-acquired pneumonia in children, showed the most significant decrease, from 48.42 to 16.97% (Table 2).

**Table 2:** Comparison of positives rates of pathogens in 2019 and 2020

Pathogens	2019 N = 1871(%)	2020 N = 725(%)	X^*2*^	P-value
Virus				
Respiratory syncytial virus (RSV)	167(8.93)	68(9.38)	0.131	0.718
Human rhinovirus (HRV)	87(4.65)	23(3.17)	2.811	0.094
Human parainfluenza virus (HPIV)	120(6.41)	21(2.90)	12.584	< 0.001
Human adenovirus (Adv)	76(4.06)	23(3.17)	1.127	0.288
Influenza A virus (FluA)	53(2.83)	14(1.93)	1.69	0.194
Influenza B virus (FluB)	15(0.80%)	11(1.52)	2.698	0.1
Bacterium				
*Streptococcus pneumoniae*	121(6.47)	40(5.52)	0.81	0.368
*Haemophilus influenzae*	131(7.00)	14(1.93)	25.475	< 0.001
*Staphylococcus aureus*	44(2.35)	47(6.48)	26.363	< 0.001
*Escherichia coli*	27(1.44)	23(3.17)	8.273	0.004
*Klebsiella pneumoniae*	37(1.98)	19(2.62)	1.024	0.312
*Pseudomonas aeruginosa*	21(1.12)	13(1.79)	1.819	0.177
Atypical pathogens				
*Mycoplasma pneumoniae*(MP)	906(48.42)	123(16.97)	216.117	< 0.001

### Changes in specific pathogens based on month

**Fig. 1:**
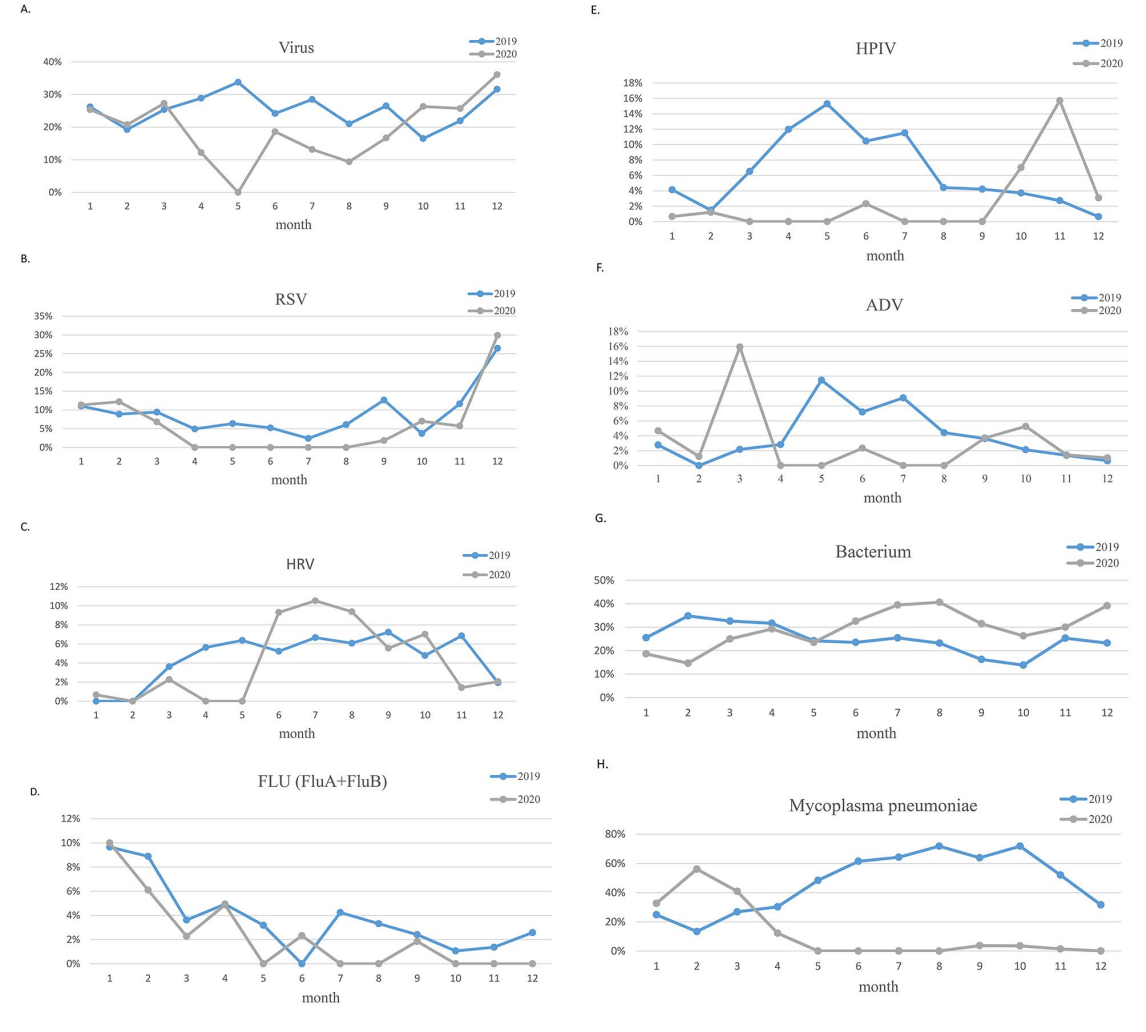
Monthly activity of pathogens during the COVID-19 pandemic year of 2020 (gray line) compared with the previous year of 2019 (blue line)

Compared with 2019, the detection rates of virus decreased after March 2020, but the seasonality in 2020 did not change, and rates also peaked in winter (Fig. 1A). RSV was at a low prevalence after April 2020, and gradually increased after October to a peak in December (Fig. 1B). In contrast to RSV, HRV increased in prevalence after schools reopened in June 2020, much higher than the same period in 2019 (Fig. 1C). Influenza had seasonal prevalence, with high incidence in winter and spring (Fig. 1D). PIV was almost undetected in the first half of 2020, with a significant increase in detection rates after September, which was opposite to the seasonal distribution in 2019 (Fig. 1E). ADV showed a small peak in early 2020, and had been in a low detection rate since then (Fig. 1F). Interestingly, circulation and seasonality of bacterium appeared to have remained the same during the two years, less affected by the COVID-19 epidemic (Fig. 1G). *Mycoplasma pneumoniae* was detected throughout the year, with a high prevalence in 2019 and peaked in autumn. Nevertheless, it was barely detected after March 2020, and low prevalence remained even after the reopening of school (Fig. 1H).

### Changes of the number of positive detections in different age groups

**Fig. 2:**
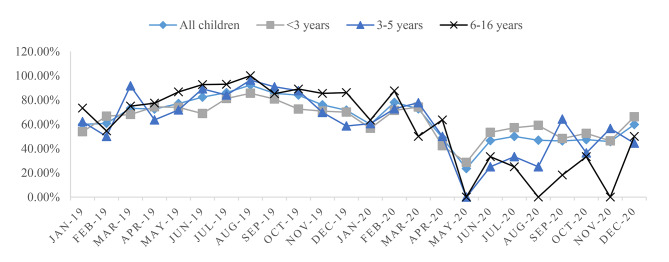
Positive detection rates of pathogens in children of different ages from January 1, 2019 to December 31, 2020

In 2019, school-age children (6–16 years) had the highest positive detection rate of common respiratory pathogens (85.25%), and the positive detection rates of < 3 years old and 3–5 years old age groups were similar (72.10%; 79.41%). Since the outbreak of the epidemic in January 2020, the detection rate of all age groups has decreased, and the most obvious was the school-age group (56.03%). However, in terms of monthly trends, the rates of positive test were similar in the three age groups (Fig. 2).

## Discussion

In this retrospective study, we assessed the epidemiological characteristics of common respiratory pathogens in children with community-acquired pneumonia before and during the local COVID19 pandemic. Our study showed an important influence of the COVID19 epidemic on the spread of common respiratory pathogens in Shanghai, China. Through a series of strict NPIs such as wearing masks, closing schools and maintaining social distance, not only the diffusion of SARS-CoV2 had been reduced, but also the epidemic pattern of other common pathogens [9], especially respiratory virus and *Mycoplasma pneumoniae*.

Compared with 2019, the number of hospitalized children with pneumonia in our department diminished by 61.25% in 2020. And the total detection rate of respiratory pathogens also fell off significantly (from 77.55 to 56.00%), whether single or mixed infection. In one research conducted in New Zealand, incidence rate of severe acute respiratory illness among hospitalized patients showed very low owing to the use of strict NPIs such as the blockade and border closures in 2020 [10]. However, our findings showed that the prevalence of pneumonia in children with congenital heart disease and the proportion of children with severe pneumonia requiring oxygen inhalation increased compared with 2019, and bacterial or RSV infection was the main cause. In terms of age, the proportion of infants had increased, probably because it was difficult for children under the age of 3 to wear masks. Perhaps, NPIs could not reduce the incidence of infants, children with underlying diseases and severe pneumonia.

Compared with 2019, the whole detection rate of viruses decreased in 2020, but the rate of RSV increased (8.93% vs. 9.38%), especially the winter peak of RSV reappeared as usual. RSV disease occurs in all age groups, but the incidence is higher under 2 years of age [11]. RSV infection has been supported to be in association with asthma and acute lower respiratory tract infection, leading mortality and morbidity to increase in children [12–14]. In this study, RSV was still the most common source of respiratory viral infection in infants (age: < 3 years), children with congenital heart disease, and severe pneumonia. Therefore, further research is needed on preventive measures for RSV. There was a small pinnacle in the positive detection rate of HPIV in the spring and summer of 2019, but it did not appear in the same period in 2020. Instead, the number of HPIV tests increased dramatically after September. Human behavior is one of the main factors influencing the seasonality infections of respiratory viruses. As a matter of fact, in the context of the easing of the domestic COVID-19 in China, people in low-risk areas have basically resumed their normal work and life, which might be the reason for the surge in RSV and HPIV infections from September to December 2020 [15]. These results showed outbreaks may take place outside of the typical season during the COVID19 pandemic. As NPIs are relaxed, it is necessary for healthcare systems to prepare for future outbreaks of ordinary respiratory viruses in children. Many studies have shown that influenza has spread in a similar way to COVID-19, such as droplet and contract transmission [16, 17]. Therefore, non-pharmaceutical interventions in linkage to reducing the spread of COVID-19 may also significantly reduce influenza [18, 19]. Despite returning to school, resumption of work and seasonal epidemics, the detection rate of 2020 influenza remained low. First, the Shanghai government increased the scope of influenza vaccination, especially for young children aged 3–6 in kindergartens in September 2020. Next, the COVID19 pandemic has changed health-seeking behavior and increased the focus on non-pharmacological interventions to decrease the risk of infection with the spread of influenza [20]. Meanwhile, many viral-viral interactions may also affect the incidence of respiratory viral infections. Interferon-stimulated immunity caused by infection with one virus can provide nonspecific interference that makes it difficult for other viruses to establish in a population [21]. Increased circulating levels of influenza A virus have been shown to limit rhinovirus epidemics, possibly through an interferon-mediated mechanism [22]. Interestingly, despite the adoption of NPIs in 2020, the detection rate of HRV increased significantly, a trend not seen with other viruses after the restarting of schools in June. A former study showed that surgical masks could keep human coronaviruses and influenza viruses from transmitting, but not rhinoviruses transmission by respiratory droplets and aerosols in symptomatic patients with acute respiratory disease [23]. In addition, rhinoviruses are non-enveloped viruses, so might be inherently less inactivated by washing hand with soap and water or by ethanol-containing disinfectant [24, 25]. Furthermore, the quality of children’s hand washing may be poor. These factors may explain the reason that rhinovirus infection remained its usual circulation level.

In terms of bacteria, the most common ones in 2019 were *Streptococcus pneumoniae* and *Haemophilus influenzae*, which were common bacteria in children with community-acquired pneumonia. Notably, Global Action Plan For Prevention and Control of Pnuemonia by the World Health Organization in 2008 listed immunization coverage for *Haemophilus influenzae* and *Streptococcus pneumoniae*, and immunization against pertussis and measles as primary prevention strategies. Given that vaccines covering for either were not routinely used in China, it was not surprising that the rates of *pneumococcal* and *Haemophilus influenzae B* infection in children were relatively high. However, by June 2020, the detection rate of bacteria increased, dominated by *Staphylococcus aureus* and *Escherichia coli*. The reason was that in the late stage of the epidemic, congenital heart disease complicated with pneumonia increased in children hospitalized in the respiratory department, whose sputum cultures were mainly *Staphylococcus aureus*, *Escherichia coli* and *Klebsiella pneumoniae*, considering with large-scale use of antibiotics, pathogenic bacteria variation, regional differences, pathogenic bacteria changes and other factors. Moreover, children with congenital heart disease are more likely to be infected with *Staphylococcus aureus* in infancy or winter than ordinary children, which may be related to factors such as their own hemodynamic characteristics and low immunity.

*Mycoplasma pneumoniae* is the one of the most popular pathogen of community-acquired pneumonia, which especially occurs in school-aged children. It can cause obvious disturbance of immune function in children. And if treatment is not timely, it will cause breathing difficulties, heart failure, etc., and even death in severe cases [26]. *Mycoplasma pneumoniae* pneumonia occurs in regional outbreaks every 3 to 7 years, and each may endure 1 to 1.5 years. The last two epidemics of MP were in 2013 and 2016 [26, 27]. When encountering epidemic years, the infection rate of MP would increase by 3 to 4 times in children and adolescents. Our study showed that the detection rate of *Mycoplasma pneumoniae* was close to 50% in 2019, based on a combination of molecular assays and serology, which was considered an outbreak of MP infection. This might also be the reason why the positive rate of school-aged children in 2019 was markedly higher than that of the other two groups of age groups. Climatic conditions, such as humidity and temperature, have been reported to affect the survival and spread of airborne *M. pneumoniae* significantly [28, 29]. 37 °C is the optimum growth temperature for MP, which grows best in the hottest months in China such as July, August and September. But in 2020 fewer patients visited clinicians following the outbreak of the COVID-19 pandemic and restrictive means against COVID-19 cut down the incidence of respiratory infections, there was a considerable reduction in the positive rate of MP since March, which remained at a comparatively low level afterwards, consistent with previous findings in other studies [30–32]. At the start of the new term, the “Guidelines for the Prevention and Control of the Novel Coronavirus Pneumonia in Primary and Secondary Schools” was issued by the Ministry of Education to give a guide and assistant on the prevention and control of the epidemic in schools. These restrictive measures on COVID-19 could effectively reduce the transmission of *Mycoplasma pneumonia*, which led to a rapid decline in the positive rate of school-aged children in 2020 as well. And it might be that older children were better able to comply with various defensive measures.

This paper not only compared the epidemiological features of common respiratory viruses in children, but also bacteria and *Mycoplasma pneumoniae* during the COVID-19 pandemic in China. However, there are some limitations in it. First, this study was conducted in a single center and all of the patients were hospitalized, which might lead to a preselection bias. Second, the methods used to detect respiratory pathogens such as viruses and bacteria were relatively simple, which might lead to false negative. Third, during the pandemic, a lot of public health interventions were enforced and some measures (such as wearing masks) still exist later. Consequently, the sample sizes should be further expanded and pathogens should be evaluated for at least two years before and after SARS-CoV2 to examine which of these measures may be the most powerful in preventing the spread of respiratory pathogens.

## Conclusions

Strict public health interventions for COVID-19 in China have effectively suppressed the spread of SARS-CoV2. We observed unprecedented reductions in Human parainfluenza virus, influenza and *Mycoplasma pneumonia*, most likely due to the role of NPIs. However, it had a much more limited effect on infants, other pathogens such as bacteria and rhinovirus. With the introduction of mass vaccination against COVID-19 and the relaxation of control measures, infection rates in younger age groups are expected to return to previous levels. Therefore, it is necessary to obtain more epidemiological surveillance of respiratory pathogens, which will help improve early preventive measures.

### Electronic supplementary material

Below is the link to the electronic supplementary material.

Supplementary Material 1

## Data Availability

The datasets generated and/or analysed during the current study are available in Supplementary Material.

## Abbreviations

ADV: Adenovirus
CAP: Community-acquired pneumonia
CP: Chlamydia pneumoniae
FluA: Influenza A virus
FluB: Influenza B virus
GICT: Immune colloidal gold technique
HPIV: Human parainfluenza virus
HRV: Human rhinovirus
MP: Mycoplasma pneumoniae
NPIs: Non-pharmaceutical interventions
PA: Particle agglutination
RSV: Respiratory syncytial virus
RT-PCR: Real-time polymerase chain reaction
SAT: Simultaneous amplification and testing
